# Isolation and characterization of a thermophilic *Streptromyces albidoflavus* strain KS5 capable of glyphosate degradation

**DOI:** 10.3389/fmicb.2026.1730482

**Published:** 2026-03-24

**Authors:** Hadjer Rebai, Cherifa Lefaida, Essam Nageh Sholkamy, Hadel El Agamawi, Mohamed A. A. Abdelhamid, Seung Pil Pack, Hazim O. Khalifa, Allaoueddine Boudemagh

**Affiliations:** 1 Department of Microbiology, Constantine 1- Frères Mentouri University, Constantine, Algeria; 2 Department of Botany and Microbiology, College of Science, King Saud University, Riyadh, Saudi Arabia; 3 Department of Civil and Environmental Engineering, United Arab Emirates University, Al Ain, United Arab Emirates; 4 Biology Department, Faculty of Education and Arts, Sohar University, Sohar, Oman; 5 Department of Botany and Microbiology, Faculty of Science, Minia University, Minia, Egypt; 6 Department of Biotechnology and Bioinformatics, Korea University, Sejong, Republic of Korea; 7 Department of Veterinary Medicine, College of Agriculture and Veterinary Medicine, United Arab Emirates University, Al Ain, United Arab Emirates; 8 Laboratory of Molecular and Cellular Biology, Constantine 1- Frères Mentouri University, Constantine, Algeria

**Keywords:** actinobacteria, ATR-FTIR, bioremediation, herbicide biodegradation, hot spring

## Abstract

A thermophilic actinobacterium was isolated from thermal waters in Khenchela, Algeria, and identified as *Streptomyces albidoflavus* (accession number: OQ727247) based on biochemical characterization and 16S rRNA gene sequencing. Designated as strain KS5, this bacterium demonstrated the capacity to degrade 50 mg L^−1^ of glyphosate within 15 days at both 30 °C and 50 °C. Remarkably, strain KS5 utilized glyphosate as its sole carbon source under these temperature conditions. Biodegradation efficiency was optimized through colorimetric assays evaluating key parameters such as pH, temperature, and inoculum size. The maximum degradation occurred at 50 °C, with optimal conditions determined at pH 7.2 and an inoculum size of 70 mg L^−1^. Total organic carbon reduction reached 53.68% at 50 °C compared to 47.27% at 30 °C. Attenuated Total Reflectance–Fourier Transform Infrared (ATR-FTIR) spectroscopy further confirmed structural modifications in glyphosate following incubation with strain KS5. Collectively, these results underscore the potential of this thermophilic *Streptomyces* strain for glyphosate biodegradation under both mesophilic and thermophilic conditions, highlighting its promise for bioremediation of environments contaminated by this herbicide.

## 1 Introduction

Glyphosate, chemically identified as *N-(phosphonomethyl)glycine*, is one of the most extensively used organophosphate herbicides worldwide, serving as a cornerstone of modern agricultural weed management practices (Li et al., 2016; Heap and Duke, 2018). Its herbicidal activity is based on the inhibition of the enzyme 5-enolpyruvylshikimate-3-phosphate synthase (EPSPS; EC 2.5.1.19), which plays an essential role in the biosynthesis of aromatic amino acids in plants and numerous microorganisms (Martins-Gomes et al., 2022). This inhibition disrupts the formation of key secondary metabolites and proteins, resulting in significant ecological consequences, including environmental contamination and potential health risks to humans (Zabalza et al., 2017).

The widespread and intensive use of glyphosate has raised growing concerns about its persistence in the environment and the urgent need for efficient degradation strategies (Maggi et al., 2020). Among the various remediation approaches, biodegradation mediated by diverse microorganisms has emerged as a sustainable and promising solution to mitigate glyphosate pollution (Zhan et al., 2018). Microbial degradation typically proceeds through two major enzymatic routes: cleavage of the carbon–phosphorus (C–P) bond by C–P lyase, and cleavage of the carbon–nitrogen (C–N) bond by N-lyase (Firdous et al., 2020). Although many microorganisms have been reported to utilize glyphosate as a sole phosphorus source during growth (Zhan et al., 2018; Firdous et al., 2020; Hernández Guijarro et al., 2021; Singh et al., 2019; Giaccio et al., 2023; Rebai et al., 2023; Fan et al., 2012; Yu et al., 2015), limited studies have focused on actinobacteria and their capacity to use glyphosate as a sole carbon source (Rebai et al., 2023; McAuliffe et al., 1990; Firdous et al., 2018). This research gap highlights the need for a deeper investigation into the metabolic pathways and molecular mechanisms by which actinobacteria mediate glyphosate degradation, thereby expanding our understanding of their biodegradative potential.

Actinobacteria are Gram-positive, filamentous bacteria that are abundant in soil ecosystems and well recognized for their ability to degrade various complex organic compounds, including pesticides (Alvarez et al., 2016; Fuentes et al., 2017; Rebai et al., 2024). Previous studies have primarily examined their activity under mesophilic conditions, as most actinobacteria exhibit reduced metabolic activity at elevated temperatures. In contrast, thermophilic bacteria, which thrive in high-temperature environments, possess specialized enzymatic and metabolic adaptations that enable efficient functioning under thermal stress. For example, *Geobacillus caldoxylosilyticus* has been shown to utilize several organophosphonates as its sole phosphorus source at 60 °C (Obojska et al., 2002), demonstrating the biotechnological potential of thermophiles for bioremediation, particularly in extreme ecosystems such as the Algerian Sahara.

The efficiency of pesticide biodegradation is governed by several environmental factors, including temperature, pH, and inoculum concentration. Hence, optimizing these parameters is crucial for maximizing microbial degradation performance (Malla et al., 2023). In this context, the present study investigates the glyphosate-degrading potential of the thermophilic actinobacterium *Streptomyces albidoflavus* under both thermophilic and mesophilic conditions, while examining the influence of key environmental parameters on the degradation process. The findings aim to enhance understanding of the biodegradation mechanisms involved and to inform the development of effective bioremediation strategies for glyphosate-contaminated environments.

## 2 Materials and methods

### 2.1 Isolation and purification of thermophilic actinobacteria

A water sample was collected from the Hammam Knif thermal spring in Khenchela, Algeria (35°29′11.63″N; 7°15′08.64″E). Samples were collected in 500 mL sterile glass vials (Baveja, 2019) and then transported to the laboratory at room temperature.

Actinobacteria were isolated using the selective medium Starch Casein Agar (SCA) (Uzel et al., 2009; Mohseni et al., 2013). The suspensions obtained were diluted serially in a sterile physiological solution up to 10^−3^, according to Bastide et al. (1986). Each dilution (0.1 mL) was then deposited on the surface of Petri dishes (Hirsch and Christensen, 1983). The cultures were incubated between 30 and 55 °C during 20–50 days. The incubator was kept in a humid atmosphere to limit evaporation and preserve the integrity of the environment.

Based on macroscopic characteristics, the isolates were purified on Bennett’s medium (Djebbah et al., 2021) and preserved at – 4 °C on agar slants of the same medium until further use. The ability of the purified strains to use glyphosate as the sole source of carbon was evaluated in mineral salt medium (MSM) containing glyphosate (CPA chem Ltd., Bulgaria. Purity 99.3%) as the sole carbon source at various concentrations (1, 10, 25, 50, 100, and 200 mg L^−1^). Plates were incubated at 30 °C during 1 week (Briceño et al., 2012). Isolates that grew at the highest herbicide concentration were then chosen for further evaluation in a liquid MSM medium supplemented with 50 mg/L of glyphosate.

### 2.2 Identification of actinobacterial isolates

The selected strain was identified by a combination of macroscopic and cultural characteristics, and a molecular identification:

#### 2.2.1 Cultural characteristics

Macroscopic and cultural properties of the selected isolate were evaluated following the guidelines of Shirling and Gottlieb (1966). The Actinobacterial strain was cultured on International *Streptomyces* Project (ISP) media, specifically ISP1, ISP2, ISP4, and ISP5. These media were employed to promote optimal growth conditions and assess the strain’s characteristics effectively (Shirling and Gottlieb, 1966). Microscopic characteristics, including morphology of aerial and substrate mycelia, spore arrangement, and Gram staining, were examined according to Cross (2009).

#### 2.2.2 Physiological and biochemical characterization

Catalase activity were assessed following the methods of Li et al. (2016), the release of gas bubbles indicates the presence of a catalase. The production of melanoid pigments was monitored according to the protocol established by Lee et al. (2014). Their formation was observed on ISP7 medium, manifested by the appearance of a black halo surrounding the colonies.

Growth tolerance to different pH values (3–11), temperatures (4, 10, 20, 30, 40, 50, 60, and 70 °C), and NaCl concentrations (1–5% w/v). The tests were conducted in flasks containing 100 mL of liquid ISP2 medium, inoculated with 1 mL of bacterial suspension. Then incubated with agitation at 120 rpm for a period of 5–10 days. Bacterial growth was assessed by measuring turbidity at 540 nm using a UV-1800A spectrophotometer (Shimadzu, Japan). The control conditions were set for all experiments at pH 7, a temperature of 30 °C, and in the absence of NaCl. Growth was assessed as positive or negative compared to a control.

#### 2.2.3 Molecular identification

Genomic DNA was extracted using a commercial extraction kit (Vivantis Technologies Sdn. Bhd., Selangor, Malaysia). PCR amplification of the 16S rRNA gene was performed using the universal primers 27F (5′-AGAGTTTGATCCTGGCTCAG-3′) and 1492R (5′-CCGTCAATTCCTTTGAGTTT-3′). PCR conditions included an initial denaturation at 94 °C for 12 min, followed by 35 cycles of denaturation at 94 °C for 30 s, annealing at 55 °C for 30 s, and extension at 72 °C for 1 min 40 s. Amplified products were resolved on a 1.5% agarose gel (Sigma-Aldrich, USA), purified using a Clean-Up kit (Vivantis), and sequenced at Apical Scientific Sdn. Bhd. (Edwards et al., 1989). The obtained 16S rRNA gene sequence was analyzed using BLAST^1^ to identify homologous sequences in the GenBank database. Phylogenetic relationships were inferred using MEGA 11 software.

### 2.3 Growth kinetics of glyphosate-degrading actinobacterium

The selected strain from qualitative screening was inoculated into MSM containing 50 mg/L glyphosate following the protocol of Briceño et al. (2012). Cultures were incubated at 50 °C under agitation 100 rpm for 21 days. Abiotic controls were incubated under identical conditions. Growth was evaluated every 3 days by measuring dry weight. All experiments were conducted in triplicate.

### 2.4 Optimization of glyphosate biodegradation conditions

To glyphosate degradation by the selected actinobacterium was evaluated under varying pH (4, 6, 7.2, and 9), temperatures (10, 25, 30, and 37 °C), and inoculum sizes based on wet biomass (20 mg L^−1^, 40 mg L^−1^, 70 mg L^−1^, and 90 mg L^−1^) according to Rebai et al. (2023). Glyphosate concentrations in the medium were quantified colorimetrically via the ninhydrin–molybdate reaction described by Bhaskara and Nagaraja (2006), and the absorbance was measured at 570 nm using a UV–visible spectrophotometer (UV-1800A, Shimadzu, Japan).

The percentage biodegradation of glyphosate was measured according to the following formula:


$$\text{Biodegradation}\;(\%)=\frac{M1-M2}{M1}\times 100\%,$$

Where *M*_1_ represents the glyphosate concentration in abiotic controls, and *M*_2_ represents that in biotic samples. Data analysis was performed using GraphPad Prism version 10.

### 2.5 Total organic carbon (TOC) analysis

The decrease in total organic carbon was measured using a Shimadzu TOC-L analyzer (Shimadzu, Kyoto, Japon) after 15 days of incubation, the samples were centrifuged, filtered, diluted, and then subjected to analysis (Rebai et al., 2024).

### 2.6 Attenuated total reflectance-fourier transform infrared (ATR-FTIR) analysis of glyphosate

Structural modifications of glyphosate during biodegradation were examined using Attenuated Total Reflectance–Fourier Transform Infrared (ATR-FTIR) spectroscopy (Thermo Fisher Scientific Inc., Madison, WI, USA). Samples of volume 1 mL extracted after 15 days of incubation were analyzed directly, after centrifugation, filtration. Spectra were recorded in the mid-infrared region (500–4,000 cm^−1^) at a scan speed of 16 (Rebai et al., 2024).

### 2.7 Statistical analysis

All experiments were conducted in triplicate, and results were expressed as mean ± standard error. Two-way analysis of variance (ANOVA) was used to evaluate the effects of the tested operational factors (pH, temperature, and inoculum size) on glyphosate biodegradation efficiency (%). When significant effects were detected, mean comparisons were performed using Tukey’s *post hoc* test at *p* ≤ 0.05. All statistical analyses were performed using GraphPad Prism version 10.

## 3 Results

### 3.1 Screening of glyphosate-degrading actinobacteria

Four strains of thermophilic bacteria (SKM, KS2, KS5, KM2) were isolated from the Hammam Knif thermal spring in Khenchela, Algeria. Among them, only strain KS5 showed a strong growth at the concentration of 50 mg L^−1^. The other strains showed a significant growth only at low concentrations (1 mg L^−1^ and 10 mg L^−1^). No growth was observed at the other concentrations tested (25, 50, 100 and 200 mg L^−1^) (Table 1).

**Table 1:** Primary screening of the growth performance of thermophilic actinobacterial isolates (SKM, KS2, KS5, KM2) under different concentrations of glyphosate as the sole carbon source.

Actinobacterial isolates	Glyphosate (mg/mL)					
	1	10	25	50	100	200
SKM	+++	++	−	−	−	−
KS2	+++	++	−	−	−	−
KS5	+++	+++	+++	+++	+	+
KM2	+++	++	−	−	−	−

(+++) strong growth, (++) medium growth, (+) weak growth, (−) no growth.

### 3.2 Biochemical, physiological, and morphological characteristics of the actinobacterial isolate

The actinobacterial isolate KS5 exhibited a catalase-positive reaction but showed no growth in any of the tested NaCl concentrations, indicating sensitivity to salinity. The strain was capable of growing across a wide pH range (4–10), with optimal growth between pH 5.5 and 7.0. It demonstrated vigorous growth at 50–55 °C, while moderate growth was observed between 20 °C and 70 °C, confirming its thermophilic nature. Furthermore, the isolate produced a distinct dark brown pigment when cultivated on ISP7 medium (Table 2).

**Table 2:** Biochemical and physiological characteristics of actinobacterial isolate KS5 on ISP7 medium at optimum conditions.

Actinobacterial isolate	Parameters				
	Temperature range (°C)	pH range	NaCl range (%)	Catalase	Melanoid pigment
KS5	50 and 55	5.5 and 7.0	−	+	+

(+) presence, (−) absence.

**Figure 1:**
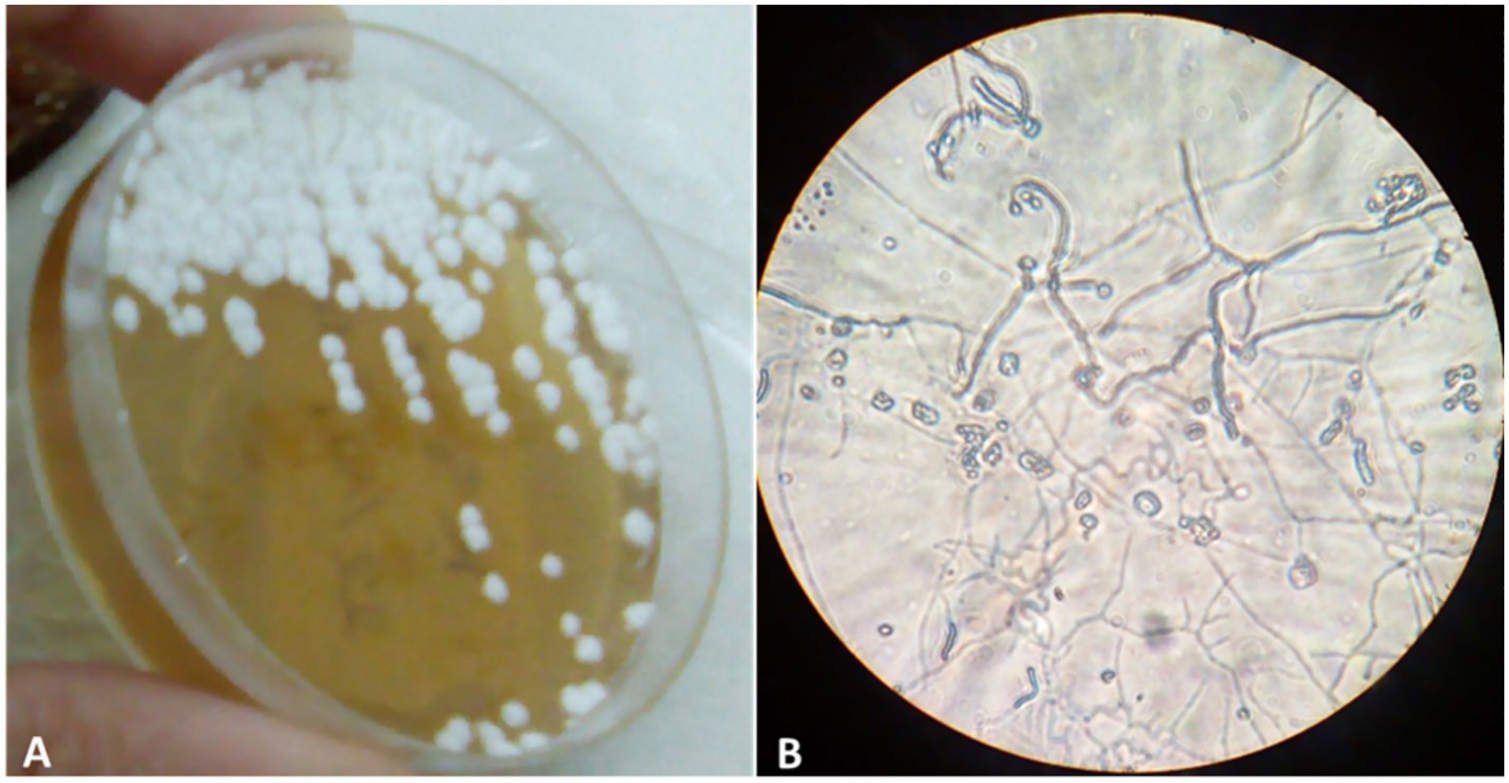
**(A)** Morphology of the actinobacterial isolate KS5 growth on ISP1 medium after 7 days; **(B)** spore chains (100× magnification).

Morphological characterization revealed that isolate KS5 formed circular, pasty colonies that adhered firmly to the agar surface. On ISP1 medium (Figure 1A), colonies developed white aerial mycelium and yellowish-white substrate mycelium. When grown on ISP2, the colonies displayed greenish-white aerial mycelium and yellowish-white substrate mycelium, consistent with its Gram-positive cell wall structure. On ISP4 medium, the isolate produced white aerial mycelium with a pale green substrate mycelium, whereas on ISP5 medium, colonies exhibited grey-green aerial mycelium with white substrate mycelium. Microscopic examination revealed that the spore chains were of the retinaculum-apertum type (Figure 1B). Growth on ISP7 medium also resulted in brown pigment production (Table 3). The isolate showed optimal growth and sporulation on ISP1, ISP2, ISP4, and ISP5 media (Table 3).

**Table 3:** Cultural characteristics of actinobacterial isolate KS5 after 15 days of incubation at 30 °C in various culture media.

Cultural characteristics	Culture medium			
	ISP1	ISP2	ISP4	ISP5
Growth	+++	+++	+++	+++
Sporulation	++	+++	++	++
Colour of aerial mycelium	White	White green	White	Grey-green
Colour of substrate mycelium	White yellow	White yellow	White green	White
Type of spore chain	Retinaculum-apertum	Retinaculum-apertum	Retinaculum-apertum	Retinaculum-apertum

(+++) strong growth, (++) weak growth.

### 3.3 Molecular identification

**Figure 2:**
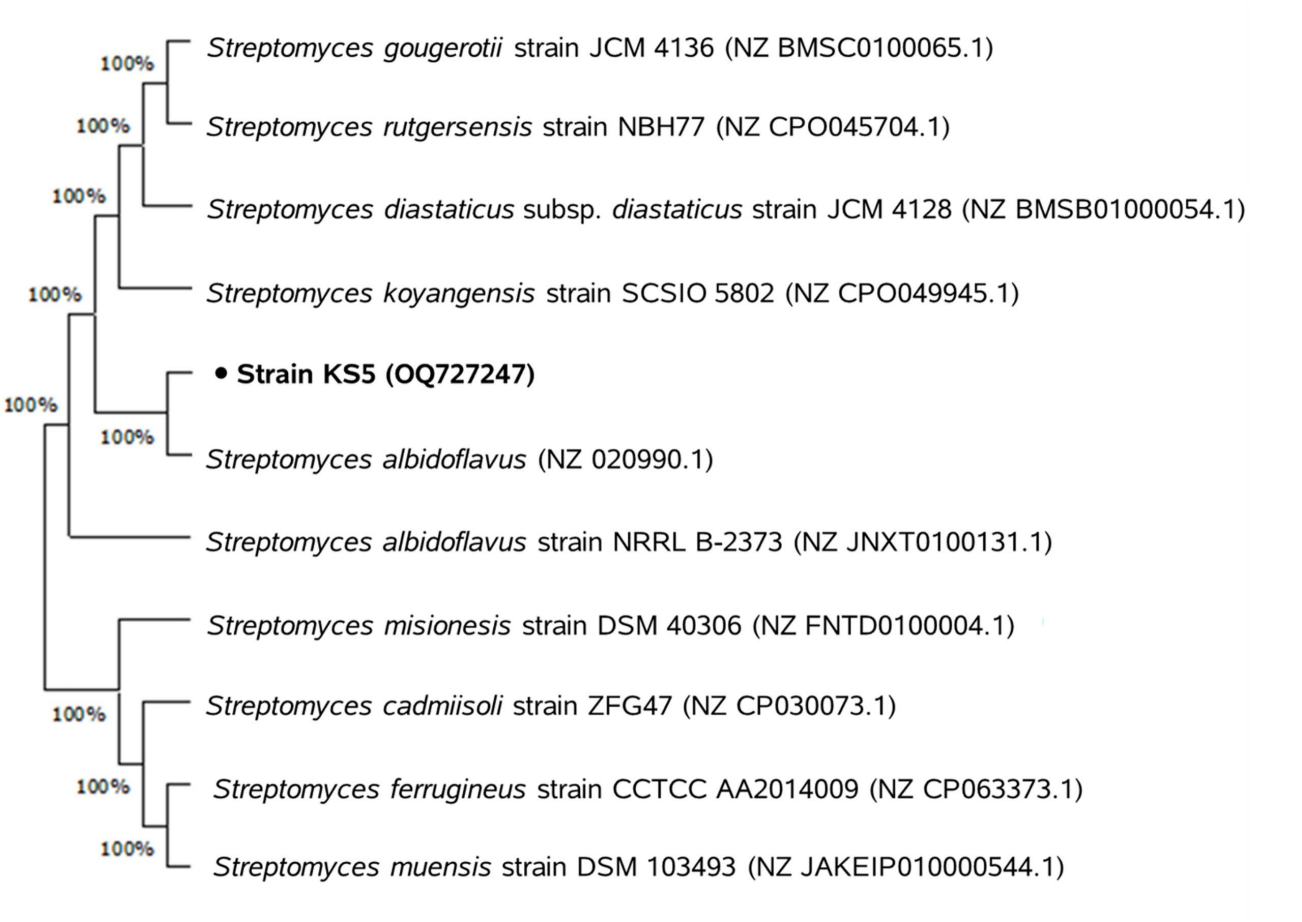
Neighbor-joining phylogenetic tree based on 16S rRNA gene sequences, showing the nearest neighbors of *Streptomyces albidoflavus* strain KS5.

The A phylogenetic tree was constructed by aligning the 16S rRNA gene sequence of the actinobacterial isolate with those of 10 *Streptomyces* reference strains retrieved from the NCBI database. The analysis revealed that the isolate shared 100% sequence similarity with *Streptomyces albidoflavus* (Figure 2), confirming its taxonomic identity.

### 3.4 Growth kinetics of glyphosate-degrading actinobacterium

**Figure 3:**
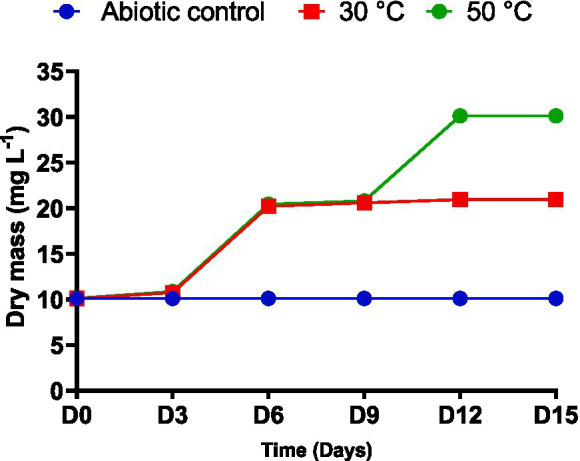
Growth curve of *Streptomyces albidoflavus* strain KS5 exposed to glyphosate concentration of 50 mg/L, along with an abiotic control at temperatures 30 °C and 50 °C.

The dry cell mass of the actinobacterial isolate was monitored every 3 days during incubation in liquid MSM medium supplemented with 50 mg L^−1^ glyphosate as the sole carbon source. As shown in Figure 3, the isolate achieved its maximum biomass on day 12, reaching 30.12 mg L^−1^ at 50 °C compared with 20.97 mg L^−1^ at 30 °C. After this point, the growth rate stabilized at both temperatures. Statistical analysis indicated no significant difference in growth between the two temperature conditions when exposed to 50 mg L^−1^ glyphosate (*p* ≥ 0.05). An abiotic control (medium without bacterial inoculation) was also included and remained stable throughout the incubation period, confirming that changes in biomass were attributable solely to microbial activity.

### 3.5 Optimization of glyphosate biodegradation conditions

**Figure 4:**
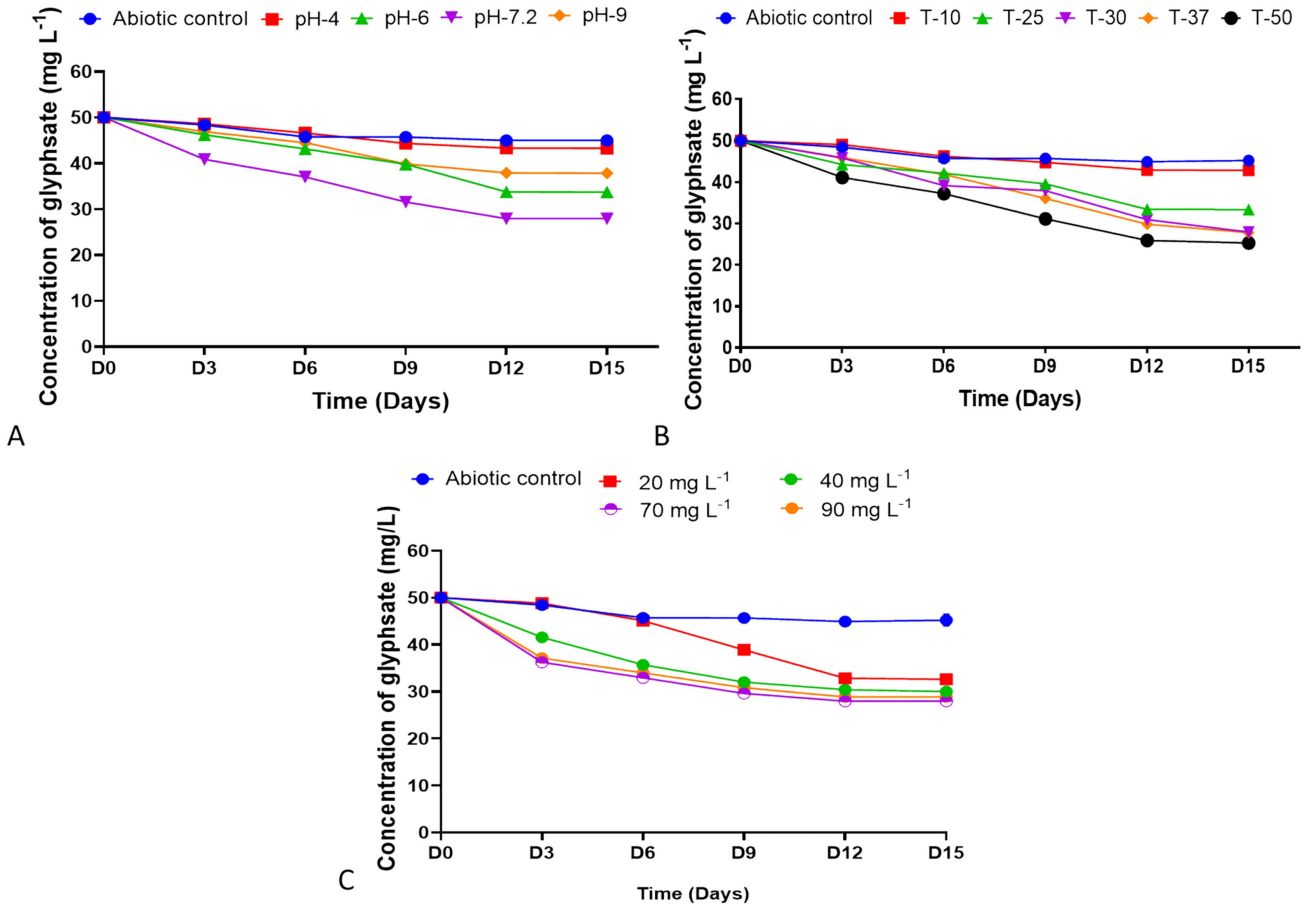
Determination of the degraded glyphosate amount by *Streptomyces albidoflavus* strain KS5 after 15 days of incubation, at **(A)** different pH, **(B)** different temperatures, and **(C)** different inoculum volumes, along with an abiotic control.

The glyphosate biodegradation by *Streptomyces albidoflavus* strain KS5 was evaluated at a concentration of 50 mg L^−1^ under varying pH, temperature, and inoculum volume conditions (Figure 4). The strain achieved its maximum degradation rate at neutral pH (7.2), with a biodegradation efficiency of 44.12%, which was significantly higher than those observed at pH 6, pH 9, and pH 4 (Tukey’s test, *p* < 0.0001). Degradation decreased to 32.58 and 24.36% at pH 6 and pH 9, respectively, and reached the lowest value of 13.46% at pH 4, confirming that pH exerted a significant influence on glyphosate degradation (*p* ≤ 0.05).

Temperature optimization revealed that the highest glyphosate biodegradation efficiency (49.38%) was achieved at 50 °C, which was significantly higher than those observed at 10 °C, 25 °C, 30 °C, and 37 °C (Tukey’s test, *p* < 0.0001). Degradation efficiencies of 44.4 and 44.12% were recorded at 37 °C and 30 °C, respectively, with no significant difference between these two temperatures (*p* > 0.05). Lower efficiencies were observed at 25 °C (33.24%) and 10 °C (14.3%), confirming that temperature had a significant effect on biodegradation efficiency (*p* ≤ 0.05).

Regarding inoculum size, the optimal degradation (44.12%) was observed with a 7% inoculum, whereas 90 mg L^−1^, 40 mg L^−1^, and 20 mg L^−1^ inoculum volumes resulted in 42.3, 39.98, and 34.8% degradation, respectively. Tukey’s *post hoc* test revealed that the degradation efficiency at 7% inoculum was significantly higher than that at 9% (*p* < 0.001), and both were significantly higher than those obtained with 40 mg L^−1^ mL and 20 mg L^−1^ inoculum volumes. These results indicate that inoculum size also exerted a significant effect on glyphosate biodegradation and overall incubation performance (p ≤ 0.05).

### 3.6 Total organic carbon (TOC) analysis

**Figure 5:**
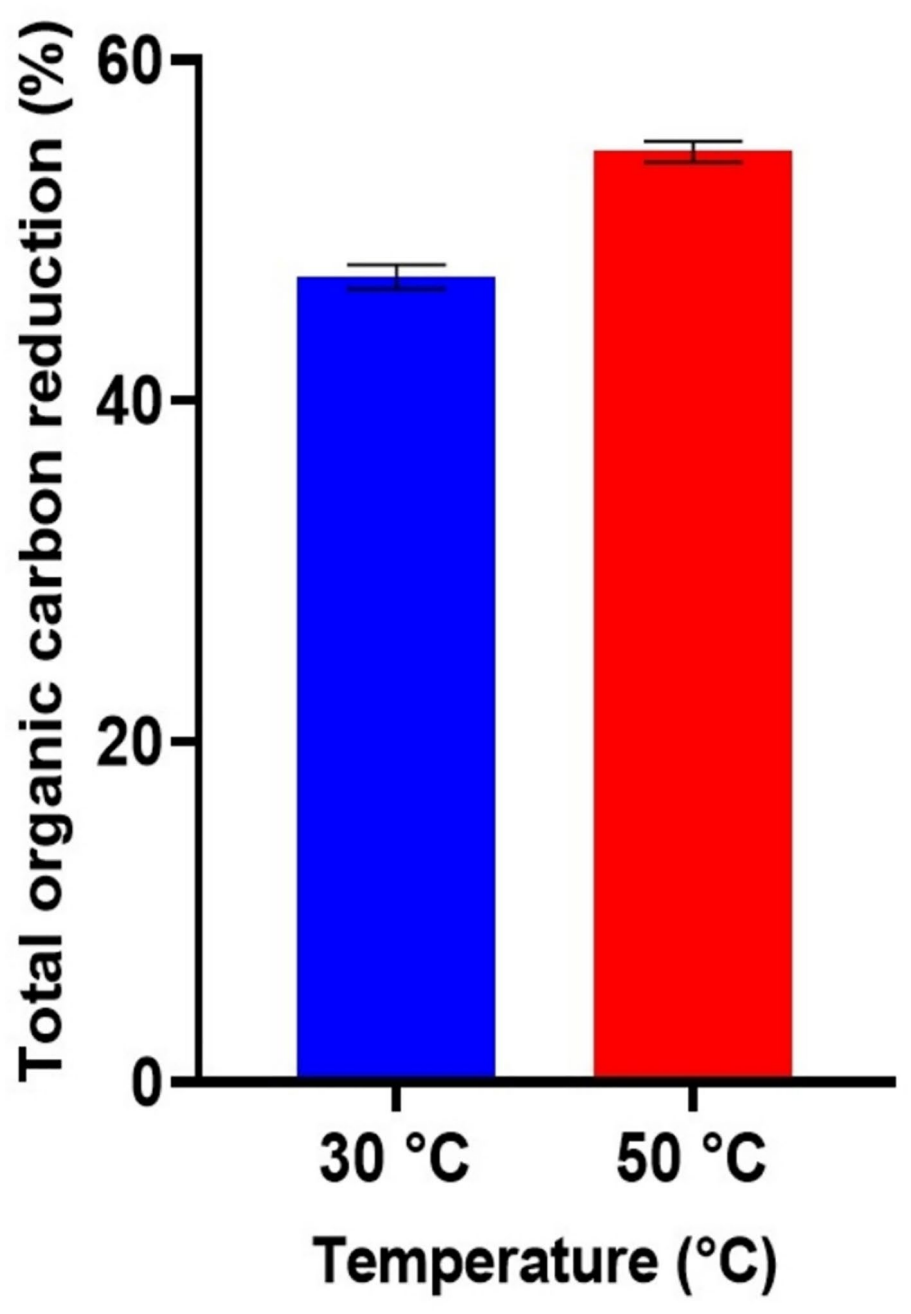
Reduction of total organic carbon TOC by *Streptomyces albidoflavus* strain KS5 during degradation of 50 mg/L glyphosate over 15 days of incubation at temperatures 30 °C and 50 °C.

The reduction of total organic carbon (TOC) during glyphosate degradation by *Streptomyces albidoflavus* strain KS5 is presented in Figure 5. The highest TOC reduction (53.68%) was recorded after 15 days of incubation at 50 °C, indicating efficient utilization of glyphosate as the sole carbon source. At 30 °C, the TOC reduction reached 47.27%, confirming that temperature influenced the mineralization efficiency of the strain.

### 3.7 ATR-FTIR analysis of glyphosate biodegradation

**Figure 6:**
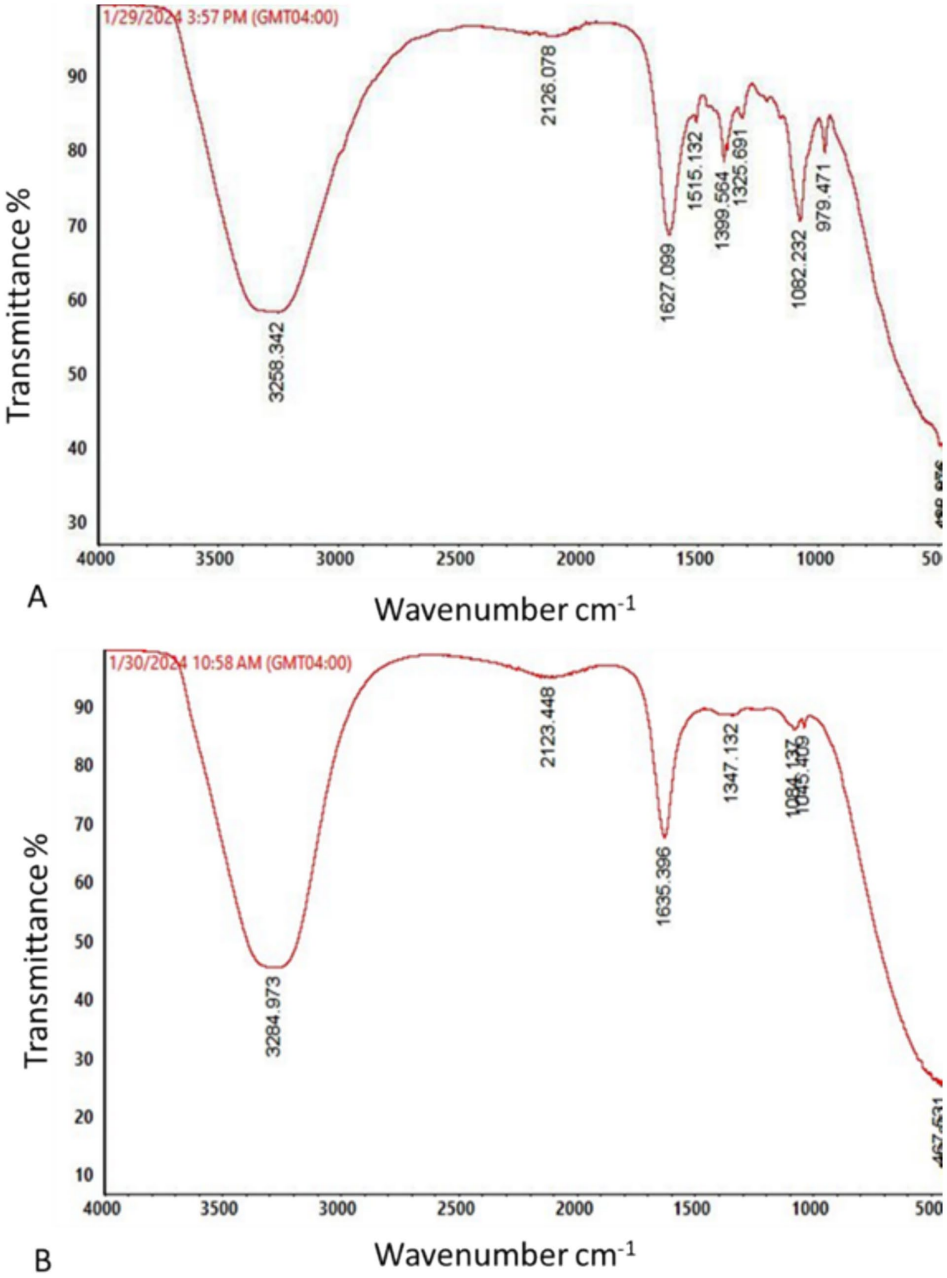
ATR-FTIR analysis of glyphosate control **(A)** and the biodegradation metabolites generated by *Streptomyces albidoflavus* strain KS5 **(B)**.

The ATR-FTIR spectra of glyphosate before and after biodegradation by strain KS5 are shown in Figure 6. Distinct spectral differences were observed between the control and the biodegraded samples, reflecting chemical alterations in the glyphosate structure. Peaks detected in the control spectrum at 979.47 cm^−1^ and 1,515.13 cm^−1^, corresponding to CH_2_ and amide group II vibrations, respectively, were absent in the spectrum of the KS5-treated sample. The disappearance of these peaks suggests cleavage or modification of these functional groups during biodegradation.

In contrast, a new absorption band at 1,045.07 cm^−1^, attributed to C–O bond stretching, appeared in the KS5 spectrum but was absent in the control. This new band indicates the formation of novel chemical groups, implying structural transformation of glyphosate resulting from microbial degradation.

## 4 Discussion

The extensive use of glyphosate, the most widely applied herbicide in modern agriculture, has raised serious environmental concerns due to its persistence and ecological toxicity. Biodegradation using microorganisms represents an effective and eco-friendly approach to mitigate glyphosate contamination (Stoyanova et al., 2023). Although numerous microorganisms have been reported to degrade glyphosate, most studies have focused on bacterial degradation under mesophilic conditions (Mohy-Ud-Din et al., 2024a; Díaz-Soto et al., 2024). In contrast, investigations involving thermophilic bacteria remain limited (Obojska et al., 2002). Given that the Algerian Sahara is characterized by high ambient temperatures that restrict the growth of mesophilic species, thermophilic microorganisms—with their ability to thrive and remain metabolically active at elevated temperatures—are of particular interest for bioremediation in such extreme environments.

Several studies have demonstrated the occurrence of actinobacteria in thermal springs, indicating their physiological adaptability to high-temperature ecosystems. For instance, *Nocardioides pakistanensis* was isolated from a thermal spring in Pakistan (Arshia et al., 2016), while *Planifilum yunnanense* sp. nov. originated from Yunnan Province, China. Similarly, *Thermoactinomyces thalpophilus* and *T. saccharis*, which grow optimally at 55 °C, were isolated from thermal spring sediments in western Anatolia, Turkey (Uzel et al., 2009). In Algeria, limited studies have addressed actinobacterial diversity in thermal environments. Medjemadj et al. (2020) identified the genera *Rathayibacter*, *Streptomyces*, and *Rhodococcus* from eastern Algerian thermal waters, while Lefaida et al. (2023) reported 13 *Streptomyces* strains from the Hammam Tleghma spring in Mila.

In the present study, the thermophilic actinobacterium *Streptomyces albidoflavus* strain KS5 was isolated from the Hammam Knif thermal spring (50 °C, pH 7.52) in Khenchela, Algeria. Phylogenetic analysis based on 16S rRNA sequences placed strain KS5 within the genus *Streptomyces*, with the closest match corresponding to *S. albidoflavus*. Although 16S rRNA provides limited resolution for definitive species-level identification in this genus, the assignment is further supported by morphological observations, including colony characteristics on ISP 1 medium and the microscopic structure of spore chains. The strain exhibited growth on MSM agar supplemented with glyphosate at varying concentrations and robust proliferation in liquid MSM medium containing 50 mg L^−1^ glyphosate, confirming its ability to utilize glyphosate as the sole carbon source. Furthermore, the colorimetric method employed to quantify glyphosate degradation proved simple, rapid, and cost-effective compared with conventional chromatographic analyses (Xu et al., 2018; Hottes et al., 2021).

Glyphosate is degraded by two major enzymes produced by bacteria: C–P lyase, which hydrolyzes the carbon-phosphorus bond (C–P), and N-lyase, which catalyzes the break of the carbon-nitrogen bond (C–N) (Firdous et al., 2020). The enzyme activity is affected by different environmental and cultivation parameters, including pH, temperature, and inoculum size. This influence highlights the need to optimize the most favorable conditions for their biodegradation (Ahmad et al., 2022).

Under optimized conditions, *S. albidoflavus* KS5 exhibited its most efficient glyphosate degradation at neutral pH and elevated temperature with a moderate inoculum size over the 12-day incubation, while the abiotic control showed only a minimal decrease in glyphosate concentration, likely due to non-biological factors associated with incubation conditions, such as light exposure or chemical interactions in the environment (Mohy-Ud-Din et al., 2024b).

The higher degradation rate at neutral pH likely reflects the strain’s adaptation to its natural environment. Similar trends have been observed in previous studies: *Comamonas odontotermitis* P2 degraded 92.2% of 1.5 g L^−1^ glyphosate at pH 7.4 (Firdous et al., 2020), and *Streptomyces* sp. strain SRH22 degraded 90.2% of 50 mg L^−1^ glyphosate at pH 7.2 (Rebai et al., 2023). Rebai et al. (2024) likewise reported optimal degradation by *Streptomyces* strains at neutral pH.

The degradation efficiency of *S. albidoflavus* KS5 increased with temperature, indicating a stronger performance under elevated thermal conditions compared to moderate temperatures where activity was lower. This result shows an adaptation of the thermophilic actinobacterium to high temperature environments. The enzymes involved in biodegradation, remarkably stable and active in this temperature range, have a favorable kinetics that facilitates the rupture of C–P and C–N bonds (Zabaloy et al., 2022). This thermophilic preference aligns with the findings of Obojska et al. (2002), who observed maximum degradation of organophosphonates by *Geobacillus caldoxylosilyticus* at 60 °C. Conversely, mesophilic bacteria often exhibit higher degradation rates at moderate temperatures; for example, *Burkholderia vietnamiensis* AQ5–12 and *Burkholderia* sp. AQ5–13 degraded 91 and 74% of glyphosate at 30 °C, respectively (Firdous et al., 2020), while *Streptomyces* strains studied by Rebai et al. (2023, 2024) also showed optimal degradation at 30 °C.

The inoculum size notably influenced degradation efficiency: at 70 mg L^−1^ inoculum, *S. albidoflavus* KS5 achieved the highest biodegradation (49.38%), with lower rates observed at smaller inoculum volumes. The initial density of the inoculum represents a determining parameter in the dynamics of biodegradation. It directly influences bacterial growth and, consequently, the enzymatic production, which determines the rate of degradation of glyphosate. A high inoculum can lead to growth limitation due to restricted nutrient availability, while an underlow inoculum exposes bacteria to disproportionate substrate concentrations, which may exert an inhibitory or toxic effect (Ramadan et al., 1990). To date, no studies have specifically addressed the effect of inoculum size on glyphosate degradation by thermophilic bacteria. However, for mesophilic strains of *Streptomyces*, Rebai et al. (2024) reported maximum degradation at 40 mg L^−1^. Lower or higher volumes reduce efficacy.

The results obtained with *Streptomyces albidoflavus* KS5 highlight its remarkable potential for the biodegradation of glyphosate in environments subject to thermal constraints. Unlike mesophilic bacteria, whose enzymatic activity decreases significantly at high temperatures, this thermophilic actinobacterium has demonstrated an ability to use glyphosate as a carbon source at both 30 °C and 50 °C. This dual performance suggests broad physiological plasticity and exceptional enzyme stability, essential characteristics for bioremediation applications in agricultural soils exposed to thermal variations. The thermostability of the enzymes involved (in particular the lyases responsible for breaking C–P and C–N bonds) gives this strain a competitive advantage under conditions where mesophilic communities become less active (Shivlata and Satyanarayana, 2015).

The observed reduction in total organic carbon (TOC) at higher incubation temperatures indicates partial mineralization of glyphosate, reflecting microbial conversion of organic carbon into inorganic end products such as CO_2_ and other mineral constituents. These values indicate that *Streptomyces albidoflavus* KS5 is not limited to a simple transformation of the pesticide into intermediate metabolites, but indeed contributes to the decrease of the organic load of the medium. The difference between the two temperatures suggests that the enzyme activity is optimized at 50 °C, which is consistent with the thermophilic nature of the strain. This partial mineralization capacity is particularly significant from a bioremediation perspective, as it reduces not only glyphosate persistence but also its potential impact on soil and water quality. However, the partial nature of the mineralization highlights the need to evaluate residual metabolites to ensure that they do not exhibit secondary toxicity (Sehrawat et al., 2021).

Few studies have reported TOC reduction by thermophilic degraders; for comparison, Korkmaz et al. (2021) observed TOC reductions of 63.04, 67.80, and 57.39% by *Bacillus aryabhattai*, *Pseudomonas azotoformans*, and *Sphingomonas pseudosanguinis*, respectively, under mesophilic conditions. Rebai et al. (2024) reported variable reductions ranging from 47.96 to 82.06% across different *Streptomyces* isolates.

ATR-FTIR spectral analyses confirmed structural modifications of glyphosate after incubation with strain KS5, including the disappearance of specific bonds and the emergence of new ones at both 30 °C and 50 °C, indicating molecular transformation during degradation. These observations are an indication of the molecular transformation of the pesticide during the biodegradation process. The loss of specific bonds suggests the breakdown of functional groups involved in glyphosate stability, while the emergence of new bands reflects the formation of intermediate metabolites or inorganic compounds. This result corroborates the TOC reduction data and reinforces the hypothesis of partial mineralization. The major interest of this spectroscopic approach is that it allows to link the enzymatic activity of the thermophilic strain with measurable chemical modifications, thus bringing an additional level of validation to the efficiency of KS5 in bioremediation. However, precise identification of the products formed remains necessary in order to assess their safety and confirm the complete detoxification of glyphosate (Korkmaz et al., 2021; Manogaran et al., 2017). Similar spectral changes were described by Rebai et al. (2024) in *Streptomyces*-mediated glyphosate degradation.

Collectively, these results demonstrate that *Streptomyces albidoflavus* strain KS5, a thermophilic actinobacterium isolated from the Algerian thermal waters, presents a remarkable potential for glyphosate biodegradation, both in mesophilic and thermophilic conditions. Its ability to use glyphosate as a carbon source and maintain high activity at 50 °C highlights its interest in bioremediation applications in thermally stressed environments, where mesophilic microorganisms are generally less performant.

Further studies are necessary to elucidate the precise enzymatic mechanisms involved in degradation, as well as to test the effectiveness of KS5 under real field conditions, including the complexity of microbial communities and abiotic factors.

## 5 Conclusion

The thermophilic actinobacterium *Streptomyces albidoflavus* strain KS5, isolated from a thermal spring in Khenchela, Algeria, exhibited a remarkable ability to degrade glyphosate and use it as a carbon source under both mesophilic (30 °C) and thermophilic (50 °C) conditions. The strain achieved a higher biodegradation rate at 50 °C (49.38%) compared with 30 °C (44.12%). Optimal degradation occurred at 50 °C, pH 7.0, and a 70 mg L^−1^ inoculum size. TOC analysis revealed substantial reductions in organic carbon at both temperatures, confirming the mineralization of glyphosate, thus reducing the risks of secondary pollution. Complementary ATR-FTIR analysis demonstrated structural alterations in the glyphosate molecule post-incubation, further validating the biodegradation process. Overall, *S. albidoflavus* strain KS5 represents a valuable biotechnological resource for the bioremediation of glyphosate-contaminated environments, particularly in thermally extreme settings where conventional mesophilic bacteria are less effective, which highlights the ecological and biotechnological relevance of thermophilic actinobacteria in bioremediation. Its thermotolerance, metabolic versatility, and efficient degradation capacity position it as a promising candidate for future large-scale bioremediation applications targeting organophosphate pollutants, and offering a solution capable of overcoming the limitations of conventional mesophilic microbes.

Additional studies are necessary to address the genetic and enzymatic pathways involved in the degradation of glyphosate by this thermophilic strain KS5, and to evaluate its ecological safety for in- situ application.

## Data Availability

The original contributions presented in the study are included in the article/supplementary material, further inquiries can be directed to the corresponding author.

## Footnotes

1
http://www.ncbi.nlm.nih.gov
